# Functional and Spatial Analysis of *C. elegans* SYG-1 and SYG-2, Orthologs of the Neph/Nephrin Cell Adhesion Module Directing Selective Synaptogenesis

**DOI:** 10.1371/journal.pone.0023598

**Published:** 2011-08-15

**Authors:** Nicola Wanner, Foteini Noutsou, Ralf Baumeister, Gerd Walz, Tobias B. Huber, Elke Neumann-Haefelin

**Affiliations:** 1 Renal Division, University Hospital Freiburg, Freiburg, Germany; 2 Bioinformatics and Molecular Genetics (Faculty of Biology) and ZBMZ Center for Biochemistry and Molecular Medicine (Faculty of Medicine), Albert-Ludwigs-Universität Freiburg, Freiburg, Germany; 3 FRIAS School of Life Sciences (LIFENET), Albert-Ludwigs-Universität Freiburg, Freiburg, Germany; 4 Centre for Biological Signalling Studies (bioss), Albert-Ludwigs-Universität Freiburg, Freiburg, Germany; 5 Spemann Graduate School of Biology and Medicine, University of Freiburg, Freiburg, Germany; Brown University, United States of America

## Abstract

The assembly of specific synaptic connections represents a prime example of cellular recognition. Members of the Ig superfamily are among the most ancient proteins represented in the genomes of both mammalian and invertebrate organisms, where they constitute a *trans*-synaptic adhesion system. The correct connectivity patterns of the highly conserved immunoglobulin superfamily proteins nephrin and Neph1 are crucial for the assembly of functional neuronal circuits and the formation of the kidney slit diaphragm, a synapse-like structure forming the filtration barrier. Here, we utilize the nematode *C. elegans* model for studying the requirements of synaptic specificity mediated by nephrin-Neph proteins. In *C. elegans*, the nephrin/Neph1 orthologs SYG-2 and SYG-1 form intercellular contacts strictly *in trans* between epithelial guidepost cells and neurons specifying the localization of synapses. We demonstrate a functional conservation between mammalian nephrin and SYG-2. Expression of nephrin effectively compensated loss of *syg-2* function in *C. elegans* and restored defective synaptic connectivity further establishing the *C. elegans* system as a valuable model for slit diaphragm proteins. Next, we investigated the effect of SYG-1 and SYG-2 *trans* homodimerization respectively. Strikingly, synapse assembly could be induced by homophilic SYG-1 but not SYG-2 binding indicating a critical role of SYG-1 intracellular signalling for morphogenetic events and pointing toward the dynamic and stochastic nature of extra- and intracellular nephrin-Neph interactions to generate reproducible patterns of synaptic connectivity.

## Introduction

A striking property of the nervous system is the precision of its uncountable numbers of synaptic connections, which are organized into specific neural circuits. Despite the diversity of mechanisms and molecules that can give rise to synaptic specificity, some important themes are beginning to emerge: Synaptic specification can operate both at the level of partner choice and at the level of synapse formation onto a specific subcellular compartment and there is now strong evidence that immunoglobulin superfamily proteins serve as molecular tags for both processes [1]. The IgSF proteins nephrin and Neph are conserved through evolution. All Neph proteins share four to five extracellular immunoglobulin-like domains and a short cytoplasmic tail that contains a conserved PDZ binding motif at the very carboxy terminus [2]. The PDZ binding motif serves as a scaffold for protein complex binding to facilitate intracellular signaling events [3]. The extracellular domains of nephrin and Neph proteins bind to each other in *cis*- and/or *trans*- interactions [4]. Two Neph1 (Roughest, Kirre) and two nephrin homologs (Hibris, Sticks-and-stones) are involved in pupal eye development and axonal guidance in *Drosophila* [5], [6], [7]. In *C. elegans,* synapse development and synaptic target recognition also involves members of the nephrin-Neph protein family. The nephrin homolog SYG-2 and the Neph1 homolog SYG-1 mediate precise recognition of appropriate partners and trigger synapse formation of the hermaphrodite specific motor neuron (HSNL) [8], [9]. The HSNL controls egg-laying behaviour by forming stereotypic *en passant* synapses on vulva muscle cells and ventral cord (VC) motor neurons. The recognition between HSNL and its targets and the precise positioning of synapses is initiated by adjacent vulva epithelial guidepost cells that express SYG-2/nephrin. SYG-2 interacts with SYG-1/Neph1 that is expressed in the HSNL, and thus recruits SYG-1 to the location along the HSNL axon where presynaptic sites are formed [8], [9].

In an exciting analogy to the nervous system nephrin and Neph molecules seem also to instruct the formation of the slit diaphragm. The slit diaphragm is a highly specialized cell-cell contact bridging the secondary foot processes of podocytes to form the most outer part of the filtrations barrier [10]. The importance of nephrin and Neph1 for the development and function of glomerular podocytes and the slit diaphragm is exemplified by the defects that have been discovered in mice and humans deficient in these molecules [11], [12], [13], [14]. Given the central role of podocytes in glomerular pathology, a lot of effort has been made to uncover the mechanisms of podocyte development, maintenance and signaling pathways. However, even though mouse models have been a useful tool in investigating podocyte functions, the mammalian glomerulus is a poorly accessible structure making it difficult to perform *in vivo* experiments. In this respect, genetically tractable model organisms such as *C. elegans, D. melanogaster* and zebrafish can overcome the difficult accessibility of slit diaphragm molecules and allow efficient functional analysis. We have previously utilized *C. elegans* to exemplify the functional complementarities between *C. elegans* SYG-1 and all three mammalian Neph proteins in regulating synaptic connectivity [15]. The asymmetric distribution of the nephrin/Neph orthologs on different cell types provides a perfect setting for further mechanistic studies of nephrin-Neph protein interactions and domain function. Here, we now show that expression of mammalian nephrin can fully compensate loss of *syg-2* function and restore synapse formation in *C. elegans*. Furthermore, we investigated the ability of SYG-1 and SYG-2, respectively, to engage homophilic interactions *in trans* between the HSN and vulva epithelial cells. Strikingly, a chimeric construct with the extracellular domain of SYG-2 and the intracellular domain of SYG-1 failed to replace full length SYG-1 in HSN, whereas full length SYG-1 was at least partially able to substitute for SYG-2 in epithelial cells.

## Results

### Expression of human nephrin can rescue defective synaptogenesis in *syg-2* mutants

**Figure 1:**
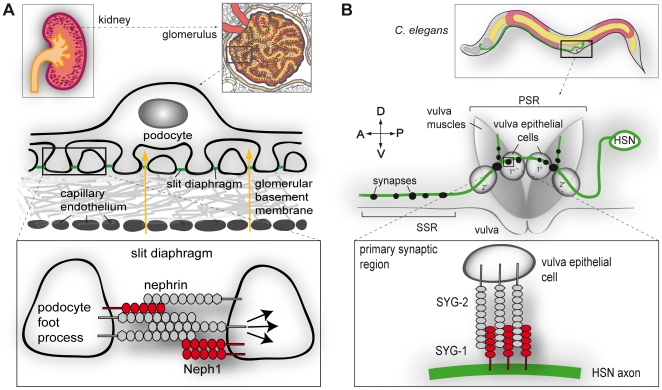
Nephrin and Neph1 are conserved across species. A, Podocytes are crucial for the filtration of blood in the kidney, together with the capillary endothelium and the glomerular basement membrane. Between their interdigitating foot processes, the podocytes form the slit diaphragm, mediated by nephrin and Neph1 *cis* and *trans* adhesion junctions, which also function as the signaling platform of the slit diaphragm (lower box). B, In *C. elegans*, the hermaphrodite specific neuron (HSN) forms synapses onto vulva muscle cells to facilitate egg laying. The specification of synapses is mediated by the vulva epithelial cells which act as guidepost cells. Adhesion of SYG-1 on the HSN to SYG-2 on vulva epithelial cells stabilizes the forming synapses in the primary synaptic region (PSR) (lower box). In *syg-1* or *syg-2* mutant worms, ectopic synapses are formed anterior of the vulva in the secondary synaptic region (SSR). Anterior (A), posterior (P), ventral (V), dorsal (D).

**Figure 2:**
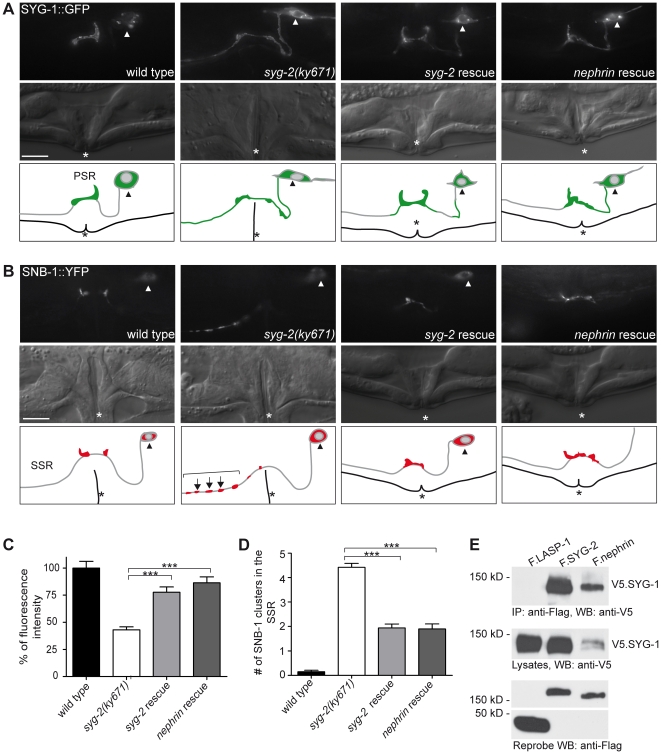
Human nephrin can rescue SYG-2 function. A, In wild type worms, the SYG-1::GFP marker is localized to the PSR, whereas in *syg-2(ky671)* mutants it is distributed all along the axon. *syg-2(ky671)* mutant animals with ectopically expressed *syg-2* or *nephrin* display enhanced SYG-1::GFP localization to the PSR. B, In wild type animals, SNB-1::YFP is localized exclusively in the PSR. In *syg-2(ky671)* mutant animals, SNB-1 clusters are visible in the SSR. *syg-2(ky671)* mutant animals with ectopically expressed *syg-2* or *nephrin* show a rescued phenotype with SNB-1 confined to the PSR. Fluorescence, DIC image and schematic drawing. Arrowhead, HSN cell body. Arrow, SNB-1 cluster. Bracket, SSR. Asteriks, vulva. Scale bar represents 10 µm. Lateral view, anterior is to the left, ventral down. C, Quantification of fluorescence intensity of SYG-1::GFP in the PSR. Ectopic expression of syg*-2* and *nephrin* rescues the *syg-2(ky671)* mutant phenotype. Mann-Whitney rank sum test. ***, p<0,001. n>19 for each strain. Error bars, SEM. D, Quantification of SNB-1 punctae in the SSR. Ectopic expression of SYG-2 and nephrin reduces the *syg-2(ky671)* mutant phenotype. Mann-Whitney rank sum test. ***, p<0,001. n>50 per strain. Error bars, SEM. E, *C. elegans* SYG-1 interacts with SYG-2 and mammalian nephrin in HEK 293T cells. V5-tagged SYG-1 and Flag-tagged SYG-2, nephrin, or LASP-1 were co-expressed in transiently transfected HEK 293T cells. After immunoprecipitation (IP) with anti-Flag antibody, the immobilized SYG-1 was detected by Western blot (WB) analysis using anti-V5 antibody (upper panel). The control protein LASP-1 failed to bind SYG-1. Middle part shows expression of V5.SYG-1 in cell lysates, the lower panel shows the expression of Flag-tagged proteins. kD, kilodalton.

We have previously reported that the Neph/nephrin family proteins can form cell-cell adhesion modules across species [15] (Fig. 1A, B). All three mammalian Neph proteins were able to restore synaptogenesis in *C. elegans* animals lacking *syg-1*. To further define the functional relationship between human nephrin and *C. elegans* SYG-2 we tested whether human nephrin is also able to rescue the *syg-2* mutant phenotype. *syg-2(ky671)* mutants have defects in SYG-1 connectivity, consequently synaptic vesicles fail to accumulate at normal synaptic locations near the vulva and instead mislocalize to ectopic secondary synaptic regions (SSR) along the HSN axon (Fig. 1B). For expression of *syg-2* and *nephrin* in adjacent vulva epithelial cells the *egl-17* promoter was used. This promoter is active in primary and secondary vulval epithelial cells [8], [16] and expression of *syg-2* under this promoter has been previously shown to result in a functional protein [9]. We constructed transgenic animals with extra-chromosomal arrays containing *syg-2* or *nephrin* driven by the *egl-17* promoter. Expression of the transgenes was confirmed by RT-PCR (Supplementary Fig. S1A). As first reporter for the functionality of nephrin and SYG-2 we used the adhesion partner SYG-1 labeled with green fluorescent protein (SYG-1::GFP). In wild type animals, SYG-1::GFP is recruited by SYG-2 and clusters exclusively to the primary synaptic region (PSR) in HSN, whereas in *syg-2(ky671)* mutant animals SYG-1::GFP fluorescence is diffusely distributed all along the axon (Fig. 2A). Strikingly, using independent transgenic lines we could show that nephrin is able to relocalize SYG-1::GFP from the axon to the contact site between the vulva epithelial cell and the HSN in *syg-2(ky671)* mutants (Fig. 2A). In all *nephrin* rescue lines we detected a significant increase of fluorescence intensity in the PSR compared to *syg-2* mutant animals (Fig. 2A and C). Interestingly, there was no difference between the *syg-2* and *nephrin* rescue lines. Furthermore, both SYG-2 and nephrin could also be shown to strongly bind to SYG-1 by co-immunoprecipitation from human embryonic kidney (HEK) 293T cells (Fig. 2E).

Presynaptic specializations in the HSN assemble within a spatially discrete location at the vulva that is stereotyped between animals [8], [17]. These presynaptic sites were visualized by the fluorophore-tagged synaptic vesicle component synaptobrevin (SNB-1::YFP) and used as a second readout for functionality of nephrin and SYG-2. This marker is confined to the PSR in wild type worms, but shows ectopic localization of several synaptic vesicle clusters anterior of the vulva in *syg-2(ky671)* mutants (Fig. 2B). Here, expression of *nephrin* rescued the synaptogenesis defect in *syg-2* mutants and restored a normal pattern of HSN synapses (Fig. 2B). Quantitative analysis revealed that the number of ectopic SNB-1::YFP synaptic vesicles in the SSR was significantly reduced in *nephrin* rescue lines compared to *syg-2* mutant animals (Fig. 2D, Supplementary Fig. S1B). Again, there was no difference between transgenic expression of *nephrin* and *syg-2*.

Together, these findings suggest that nephrin can recruit SYG-1 to an appropriate location on the HSN and induce synapse formation indicating functional complementarity between *C. elegans* SYG-2 and mammalian nephrin in the formation of cell-cell contacts and the assembly of primary synapse regions.

### SYG-1 function cannot be rescued by homotypic trans-interaction of SYG-2

**Figure 3:**
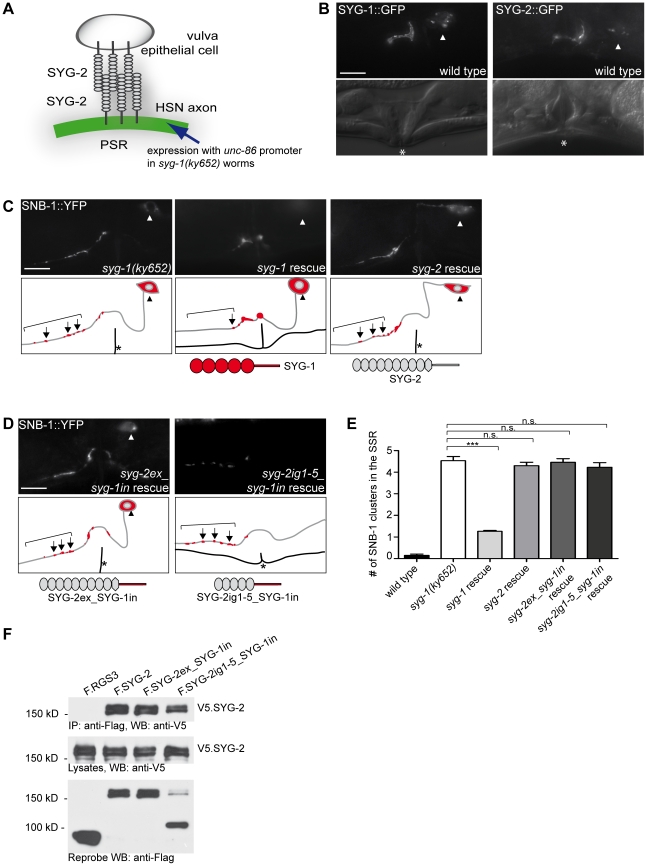
SYG-2 cannot rescue SYG-1 function in HSN. A, Schematic representation of SYG-2 transgene expression in the HSN under the *unc-86* promoter. B, SYG-1::GFP expressed in the HSN localizes to the vulva area in wild type worms. Under the same promoter, SYG-2::GFP also localizes to the vulva area. Fluorescence and DIC image. C, *syg-1(ky652)* mutant animals display SNB-1::YFP vesicle clusters in the SSR. Ectopic expression of *syg-1* in the HSN of *syg-1(ky652)* mutants rescues the mutant phenotype. *syg-1(ky652)* mutants with ectopic expression of *syg-2* in the HSN, however, display synaptic vesicle clusters in the SSR. D, *syg-1(ky652)* mutants with ectopic expression of *syg-2ex_syg-1in* and *syg-2ig1-5_syg-1in* in the HSN also display synaptic vesicle clusters in the SSR. Fluorescence image and schematic drawing. Arrowhead, HSN cell body. Arrow, SNB-1 cluster. Asteriks, vulva. Scale bar represents 10 µm. Lateral view, anterior is to the left, ventral down. E, Quantification of SNB-1 punctae in the SSR. Ectopic expression of *syg-2*, *syg-2ex_syg-1in*, and *syg-2ig1-5_syg-1in* does not reduce the *syg-1(ky652)* mutant phenotype contrary to expression of *syg-1*. Mann-Whitney rank sum test. ***, p<0,001. n.s., not significant. n>30. Error bars, SEM. F, SYG-2 interacts with SYG-2 and chimerical SYG-2_SYG-1 proteins. V5.SYG-2 and Flag-tagged SYG-2, SYG-2ex_SYG-1in, SYG-2ig1-5_SYG-1in, or RGS3 were expressed in transiently transfected HEK 293T cells. After immunoprescipitation (IP) with anti-Flag antibody, the immobilized SYG-2 was detected with anti-V5 antibody in the precipitates containing SYG-2, SYG-2ex_SYG-1in, SYG-2ig1-5_SYG-1in, but not RGS3 (upper panel). The middle panel shows expression of V5.SYG-2 in the lysates, the lower panel expression of the Flag-tagged proteins. kD, kilodalton.

The distribution of nephrin and the Neph proteins in podocyte foot processes is symmetrical across the slit diaphragm, meaning that all proteins are expressed in both adjacent podocytes (Fig. 1A). While nephrin *trans*-homodimer formation is crucial for the integrity of the slit diaphragm, *cis*- and *trans*-heterodimers with Neph1 have also been implicated in important signaling functions at the slit diaphragm [4], [11], [18]. Contrarily, in *C. elegans* the expression of the nephrin/Neph1 orthologs SYG-2 and SYG-1 is clearly separated into vulva epithelial cells and HSN (Fig. 1B). We used this asymmetric distribution to explore homophilic *trans*-interaction of SYG-2 analogous to its ortholog nephrin. Therefore, we expressed *syg-2* under the control of the *unc-86* promoter in the HSN of *syg-1(ky652)* mutant animals (Fig. 3A) [19]. When visualized by GFP reporter gene fusion, SYG-2 localized to the PSR just like SYG-1::GFP (Fig. 3B). However, SYG-2 could not rescue the synaptogenesis defect of the *syg-1(ky652)* mutant, contrary to the rescue with SYG-1 (Fig. 3C) [8]. SNB-1::YFP stained synaptic vesicles were still mislocalized anterior of the vulva (Fig. 3C). Quantification of the number of ectopic synapses in the SSR revealed no significant difference between either *syg-2* transgenic line and the *syg-1* mutant animals (Fig. 3E, Supplementary Fig. S1C). However, strong homodimerzation of SYG-2 could be shown in co-immunoprecipitation in HEK 293T cells (Fig. 3F). This lack of compensation might be explained by the importance of the intracellular domain of SYG-1. Intracellular binding of SYG-1 to SKR-1, a *C. elegans* homolog of a core component (*Skp1*) of the SCF complex, has been shown to be involved in protection of synapses in the PSR [20].

We therefore replaced the intracellular domain of SYG-2 with that of SYG-1 and tested this chimerical *syg-2ex_syg-1in* construct for synapse formation. Still, there was no rescue of the *syg-1(ky652)* mutant phenotype (Fig. 3D). Considering the length of SYG-2 with nine immunoglobulin domains compared to five in SYG-1, the space formed by adhering SYG-2 molecules between the opposing cells might be wider, maybe impairing synapse formation at the PSR. To test this possibility, we created a chimerical construct with the first five immunoglobulin domains of SYG-2 and the intracellular domains of SYG-1 (SYG-2ig1-5_SYG-1in). However, this construct failed to rescue synaptogenesis as well (Fig. 3D). There was no significant difference in the number of ectopic synapses at the SSR between either transgenic line and the *syg-1* mutant animals (Fig. 3E, Supplementary Fig. S1C). We also tested the binding of SYG-2 to both chimerical constructs, SYG-2ex_SYG-1in and SYG-2ig1-5_SYG-1in. In all cases, strong adhesion could be shown by co-immunoprecipitation in HEK 293T cells (Fig. 3F). Although localizing to the appropriate subcellular plasma membrane area in the HSN and binding to SYG-2, all constructs failed to provide the necessary intracellular signaling for the specification of synapses.

### SYG-2 function can be partially compensated by homotypic trans-interaction of SYG-1

**Figure 4:**
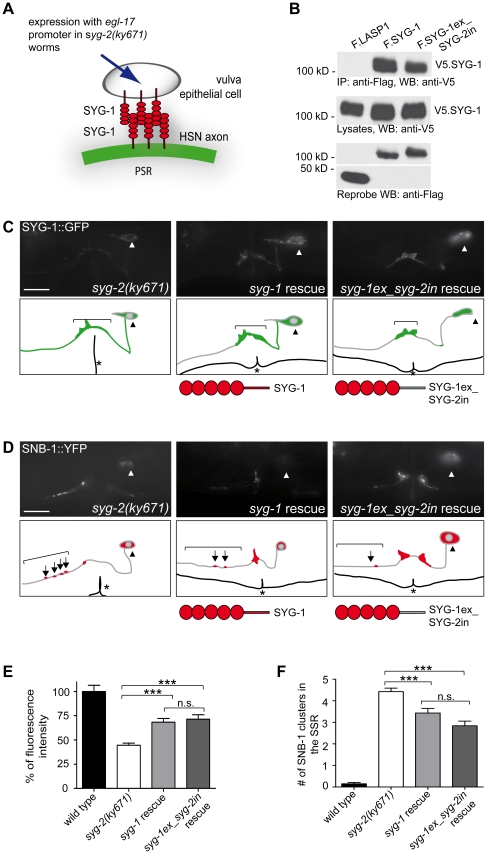
SYG-1 can partially rescue SYG-2 functions in vulva epithelial cells. A, Schematic representation of SYG-1 transgene expression in the vulva epithelial ecells under the *egl-17* promoter. B, SYG-1 interacts with SYG-1 and chimerical SYG-1ex_SYG-2in. V5-tagged SYG-1 and Flag-tagged SYG-1, SYG-1ex_SYG-2in, or LASP1 were co-expressed in HEK 293T cells and precipitated with anti-Flag antibody. V5.SYG-1 bound to SYG-1 and chimerical SYG-1ex_SYG-2in was detected using anti-V5 antibody The control protein LASP-1 failed to immobilize SYG-1 (upper panel). The middle panel shows expression of V5.SYG-1 in the lysates, the lower panel expression of the Flag-tagged proteins. IP, immunoprecipitation. WB, Western blot. kD, kilodalton. C, Ectopically expressed *syg-1* and *syg-1ex_syg-2in* in *syg-2(ky671)* mutants increases localization of SYG-1::GFP to the PSR in HSN compared to mutant animals. D, Ectopic expression of *syg-1* and *syg-1ex_syg-2in* in vulva epithelial cell of *syg-2(ky671)* mutants reduces SNB-1 clusters in the SSR. Fluorescence image and schematic drawing. Arrowhead, HSN cell body. Arrow, SNB-1 cluster. Asteriks, vulva. Scale bar represents 10 µm. Lateral view, anterior is to the left, ventral down. E, Quantification of fluorescence intensity of SYG-1::GFP in the PSR. Ectopic expression of *syg-1* and *syg-1ex_syg-2in* partially rescues the *syg-2(ky671)* mutant phenotype. Student t-test/Mann-Whitney rank sum test. ***, p<0,001. n.s., not significant. n>20. Error bars, SEM. F, Quantification of SNB-1::YFP punctae in the SSR. Ectopic expression of *syg-1* or *syg-1ex_syg-2in* partially reduces the *syg-2(ky671)* mutant phenotype. Mann-Whitney rank sum test. ***, p<0.001. n.s., not significant. n>50. Error bars, SEM.

Following the *trans*-interaction of SYG-2, we also tested the ability of SYG-1 to form *trans* homodimers between HSN and vulva epithelial cells. For this purpose, we expressed *syg-1* under the *egl-17* promoter in the secondary vulva epithelial cells of *syg-2* mutants (Fig. 4A). Using SYG-1 fused to GFP as a marker for adhesion between the HSN and vulva epithelial cells, a significant redistribution of SYG-1::GFP intensity towards the PSR could be seen, indicating that transgenic SYG-1 on vulva epithelial cells successfully mediated the adhesion to SYG-1 on the HSN (Fig. 4C and E). Furthermore, the quantification of the number of SNB-1::YFP stained synapses showed a significant reduction of ectopic vesicle clusters in the *syg-1* rescue lines compared to *syg-2* mutants (Fig. 4D and F). These findings suggest that homotypic *trans*-interactions of SYG-1 on vulva epithelial cells and HSN are sufficient to drive synapse formation. Interestingly, the rescue of the *syg-2* mutant phenotype achieved with SYG-1 did not reach the power of the SYG-2 or nephrin rescue.

Next, we wondered if the intracellular domain of SYG-2 can improve rescue properties of SYG-1. Changing the intracellular domain of SYG-1 with that of SYG-2, we obtained similar results both with SYG-1::GFP and SNB-1::YFP synaptic vesicle marker (Fig. 4C and D). Quantified, the fluorescence intensity of SYG-1::GFP differed significantly between *syg-1ex_syg-2in* rescue lines and *syg-2(ky671)* mutant (Fig. 4E). Moreover, the number of synapses in the SSR showed a significant reduction of vesicle clusters in the *syg-1ex_syg-2in* rescue lines compared to the *syg-2* mutant (Fig. 4F). Here, the rescue achieved with the chimerical *syg-1ex_syg-2in* transgene was not significantly improved compared to the *syg-1* rescue line (Fig. 4E and F). The only noted difference was slightly more worms with a full-blown mutant phenotype of four or more SNB-1 clusters in the SSR in the *syg-1* rescue worms compared to the *syg-1ex_syg-2in* transgenic animals (Supplementary Fig. S1D). The interaction of SYG-1 and SYG-1ex_SYG-2in with SYG-1 could be verified by co-immunoprecipitation in HEK 293T cells (Fig. 4B). Together, these findings indicate that the extracellular adhesion properties of the immunoglobulin membrane proteins play a crucial role for the assembly of the primary synapse region of the HSN. Intracellular signaling functions in the vulva epithelial cells might to a lesser extend influence synapse formation in the HSN.

## Discussion

The slit diaphragm represents the most vulnerable structure of the ultrafiltration apparatus in the kidney. Regulatory mechanisms influencing the formation, differentiation, and maintenance of the slit diaphragm are required to ensure glomerular filtration [21]. The immunoglobulin superfamily proteins nephrin and Neph1 form the core of the slit diaphragm. Together these proteins compose a cell-cell recognition module which is highly conserved across species. We exploited this conservation to study the fundamental mechanistic principles of the nephrin/SYG-2 and Neph1/SYG-1 interplay using the *C. elegans* HSN synapses as a model. The present study expands upon previous work showing that all three mammalian Neph proteins are able to partially rescue the synaptic developmental defects of *syg-1* mutants [22]. Here, we analyzed the capacity of nephrin to functionally compensate for the loss of *syg-2*. Strikingly, heterologous expression of *nephrin* in vulva epithelial cells was able to relocalize SYG-1 to appropriate sites on HSN and drive synapse formation in *syg-2* mutants. The full rescue of the synaptic phenotype of *syg-2* mutants underlines the high level of conservation of nephrin molecules throughout evolution and confirms the ability of these adhesive molecules in inducing morphogenetic processes in a different cellular context. Recently, it was shown that nephrin is highly expressed in the mouse central nervous system and localizes to synapses [23]. This expression pattern and the interaction with neurotransmission receptors and synaptic scaffolding molecules might point to a possible role for the mammalian nephrin adhesion complex in synapse formation, but the precise function still needs to be clarified.

In this study, we further performed structure-function analyses on SYG-1 and SYG-2. In *C. elegans,* SYG-2 and SYG-1 are asymmetrically located on different cell types and their interactions seem to appear only *in trans*, whereas nephrin has been shown to be able to form *trans* homo- and heterotypic interactions with molecules on adjacent podocyte foot processes. Thus, the *C. elegans* system is exceptionally well suited for functional and spatial analyses and we utilized this *in vivo* model to investigate the effects of *trans* homotypic SYG-2 adhesions. We demonstrated that expression of full length *syg-2* in HSN was unable to rescue the *syg-1* mutant phenotype although the transgene was tethered to the right plasma membrane area. These findings indicate that contact-mediated mechanisms are not sufficient to trigger HSN synapses and underline the fundamental importance of SYG-1 intracellular signaling pathways for synapse assembly. Notably, binding of SYG-1 to SKR-1 has been shown to block ubiquitination activity by the SCF E3 ligase complex and resulted in protection of synapses in the PSR [20]. Moreover, synaptogenesis in *syg-1* mutants could not be restored by a chimerical protein with the intracellular tail of SYG-1 and the extracellular domain of SYG-2. A shorter construct with only the first five immunoglobulin domains of SYG-2 and the intracellular tail of SYG-1 achieved no reduction of ectopic synapses either. Of note, previous studies demonstrated that the N-terminal Ig domains 1-5 of SYG-2 are critical for *syg-2* function in epithelial cells [24]. The length of 5 SYG-2 Ig-domains in this construct resembles exactly extracellular SYG-1 and therefore should appropriately span the contact between HSN and the vulva epithelial cell. Nonetheless, our biochemical results illustrated that all transgenic constructs were able to bind to SYG-2 *in vitro*. Our results suggest that the mechanisms involved in SYG-1 adhesion and intracellular signaling are very delicate: To ensure a functional rescue of the immunoglobulin protein SYG-1 the transgenic molecule needs to contact the physiological ligand, requires to localize it to the predetermined membrane site of future synapses and has to promote intracellular signaling. Furthermore, SYG-1 interaction with an extra- or intracellular scaffold might enhance the recruitment of synaptic components.

We subsequently examined the analogous homotypic *in vivo* adhesion functions of SYG-1 in the *C. elegans* system. In contrast to nephrin, the SYG-1 homolog Neph1 is believed to form *cis* but not *trans* homodimers at the slit diaphragm due to its length [4]. Remarkably, expression of SYG-1 *in trans*, in vulva epithelial cells, resulted in a significant reduction of mislocalized synapses, indicating a partial rescue of the *syg-2* mutant phenotype. Interestingly, SYG-1 was less effective than SYG-2 in restoring synaptogenesis. Corresponding experiments were recently done in a *Drosophila* S2 cell culture system with the *C. elegans* and *D. melanogaster* orthologs of nephrin and Neph1. Shen *et al*. transfected S2 cells with either SYG-1 or SYG-2. Contrarily to our results, which show that SYG-1 homophilic interaction can guide HSN synapse localization, in this model cells only aggregated when they could form heterophilic interactions [9]. However, *Drosophila* Neph1 orthologs Duf/Kirre could engage in homotypic cell adhesion and led to S2 cell aggregation. Again, heterotypic interactions between SNS and Duf/Kirrre occured more rapidly and stably than homotypic interactions [25]. The consensus of all studies, including ours, is that heterotypic interactions between the nephrin/Neph1 orthologs form the strongest bonds. Consequently, the functional specificity of SYG-1 and SYG-2 also resides in their extracellular Ig domains. Local strength of adhesion between the molecules on opposed membranes might determine synapse specification. Analysis of cell adhesion driven by Cadherin provided evidence that surface concentration and strength of affinity impacts synapse formation [26]. Moreover, recruitment of additional proteins to the SYG-1/SYG-2 cell adhesion complex may be necessary for stabilization of the complex, directing target recognition, regulating differentiation of pre- and postsynaptic specializations or modulation of synaptic structure and function. There is ample evidence that IgSF can engage in a variety of different interactions to form macromolecular complexes either via their extracellular domains or cytoplasmic tails thus performing localized signaling or adhesive functions. Extracellular matrix proteins and secreted molecules have been shown to affect assembly of synaptic sites. Very recently OIG-4, a secreted Ig protein, was demonstrated to regulate the stability of the acetylcholine receptor complex [27]. Laminin, a basement membrane component binds to calcium channels and leads to assembly of presynaptic terminals at the neuromuscular junction [28]. Intracellularly, a number of scaffolding proteins are thought to organize presynaptic sites. For example liprin-α/SYD-2 has been shown to regulate size of active zones downstream of *syg-1* [29].

Although homophilic SYG-1 interaction can target HSN synapses, SYG-1 was less effective than SYG-2. Furthermore, a chimeric construct combining the extracellular Ig domains of SYG-1 and the intracellular tail of SYG-2 did not affect the extend of the rescue. This observation may reflect that SYG-2 functions in epithelial guidepost cells are mostly based on recognizing and immobilizing SYG-1 ligand. A complementary study by Chao demonstrated that the highly conserved PDZ-binding motif of SYG-2 was dispensable for *syg-2* function as expression of *syg-2* lacking the PDZ motif efficiently rescued *syg-2* mutants [24]. Together, this might indeed be a great difference to nephrin function at the slit diaphragm, where nephrin signaling is required for several podocyte cell functions including cytoskeletal organization.

In past years the role of the podocytes as the central target of glomerular diseases has become increasingly evident. Knowledge about intracellular mechanisms of podocyte injury leading to the progression of renal failure will hopefully lead to the identification of novel therapeutic targets. Due to the inaccessibility of the slit diaphragm, novel approaches and model systems will be crucial for the investigation of slit diaphragm molecule function.

Based on the high compatibility of nephrin/Neph1 function in the *C. elegans* model system we could identify the mechanisms of SYG-1/SYG-2 homotypic binding properties and intracellular functions and identify the HSN *C. elegans* model as a powerful tool to study the function of mammalian nephrin/Neph1 molecules *in vivo*.

## Materials and Methods

### Strains

Wildtype N2 Bristol, *kyIs288 [Punc-86::syg-1::gfp;Podr-1::dsred], kyIs235 [Punc-86::snb-1::yfp;Punc-4::lin-10::dsred;Podr-1::dsred]*, *syg-1(ky652);kyIs235, syg-2(ky671);kyIs235, syg-2(ky671);kyIs288.* Maintenance of strains was according to standard methods at 15, 20 or 23°C [30].

### Transgenic lines

*syg-2(ky671);kyIs235;Ex[Pegl-17::syg-2], syg-2(ky671);kyIs235;Ex[Pegl-17::nephrin], syg-2(ky671);kyIs288;Ex[Pegl-17::syg-2], syg-2(ky671);kyIs288;Ex[Pegl-17::nephrin], Ex[Punc-86::syg-2::gfp], syg-1(ky652);kyIs235;Ex[Punc-86::syg-2], syg-1(ky652);kyIs235;Ex[Punc-86::syg-2Ex_syg-1In], syg-1(ky652);kyIs235;Ex[Punc-86::syg-1Ig1-5_syg-2In], syg-2(ky671);kyIs235;Ex[Pegl-17::syg-1], syg-2(ky671);kyIs288;Ex[Pegl-17::syg-1], syg-2(ky671);kyIs235;Ex[Pegl-17::syg-1Ex_syg-2In], syg-2(ky671);kyIs288;Ex[Pegl-17::syg-1Ex_syg-2In].*

### Molecular biology

For the expression of human nephrin and SYG-2 under the *egl-17* promoter in *C. elegans* secondary vulva epithelial cells full length cDNA was amplified by PCR and cloned with *MluI/NotI* restriction sites into a pPD95.75 vector containing 3.5 kb *egl-17* promoter region. For the expression of constructs under the *unc-86* promoter in the HSN the constructs were cloned with *NheI/NcoI* restriction sites into a pSM vector. GFP-tagged constructs were cloned without stop codon in pSM [8]. Chimeric constructs were cloned using additional restriction sites (*syg-2ex_syg-1in, syg-2ig1-5_syg-1in*) or overlapping PCR fragments (*syg-1ex_syg-2in*).

SYG-2ex_SYG-1in: amino acids 1-1083 of SYG-2 and amino acids 552-730 of SYG-1.

SYG-2ig1-5_SYG-1in: amino acids 1-582 of SYG-2 and amino acids 552-730 of SYG-1.

SYG-1ex_SYG-2in: amino acids 1-574 of SYG-1 and amino acids 1084-1270 of SYG-2.

The domains were determined with SMART (http://smart.embl-heidelberg.de) and according to Chao [24]. All construct were sequenced prior to microinjection.

Constructs for co-immunoprecipitation were created by fusing the cDNAs or chimeric constructs without signaling sequence to a 3′ V5- or Flag-tag containing pcDNA6 vector with the signaling peptide sequence of CD5 [3]. The signaling peptides were determined with SignalP 3.0 (http://www.cbs.dtu.dk/services/SignalP). For SYG-1 amino acids 1–18, for SYG-2 amino acids 1–19 were identified as the signaling peptides.

### RNA isolation and RT-PCR

A Qiagen RNeasy kit was used following the manufacturers' guidelines to isolate RNA from approximately 100 young adult worms per strain. cDNA was synthesized with Superscript III Reverse Transcriptase (Invitrogen) and oligodT primers. The following primer pairs were used for RT-PCR:

*syg-2* fp: CAGATCTATTAATGCCAGAG, *syg-2* rp: GATAATCGTATTCCAGTAGG. *nephrin* fp: CCTGTGCTGTTCGCTCTTGG, *nephrin* rp: TCTTCAGGCCAGTGGAGGTC. *act-1* fp: ATGTGTGACGACGAGGTTGC, *act-1* rp: TAGATTGGGACGGTGTGGGT.

The PCR products were run on an agarose gel with ethidiumbromide.

### Transgenic lines and fluorescence microscopy

Transgenic lines were created by microinjection of DNA plasmids into the gonads of young adult worms at a concentration of 5 ng/µl, together with 10 ng/µl of the co-injection marker *Pmyo-2::gfp* (pBY1999) or 20 ng/µl pRF4 (*rol-6(su1006)*). Several independent transgenic lines were examined for the SNB-1::YFP or SYG-1::GFP phenotype. Representative lines were chosen for quantification.

SNB-1::YFP clusters in the PSR were analyzed in young adult worms with a Zeiss Axiophot 2 microscope.

SYG-1::GFP intensity was analyzed with a Zeiss Axiovert 200 M microscope and AxioCam camera. PSR fluorescence intensity of the images was measured with ImageJ. Background intensity was subtracted. Fluorescence intensity of the wild type was set to 100%.

Fluorescence and DIC images for the publication were taken with a Zeiss AxioImager Z1 microscope.

### Cell culture and transfection

For co-immunoprecipitations, HEK 293T cells (received from American Type Culture Collection, ATCC, Manassas, VA) were grown in DMEM with 10% FBS. The 70–90% confluent cells were split at a ratio of 1∶5 and transfected the next day with plasmid DNA applying the calcium phosphate method as described previously [31]. The transfection was stopped after 6–8 h by replacing the media.

### Co-immunoprecipitation

The cells were harvested the next day in PBS and lysed in lysis buffer containing 1% Triton X-100. The supernatant was centrifuged at 15,000 xg (15 min, 4°C) and 100,000 xg (30 min, 4°C) and then incubated for 1–2 h with M2-beads. The beads were washed four times with lysis buffer and then incubated at 95°C for 5 min. The proteins were separated by a 7.5% SDS-PAGE and the Western blot stained with anti-V5 mAB (Serotec) or anti-Flag M2 mAB (Sigma) and anti-mouse Ig HRP-coupled pAB (Dako). The membranes were stripped with buffer containing 2% SDS and 100 mM beta-mercaptoethanol at 50°C for 15 min before the second staining.

### Statistical analysis

Statisical analysis was done using SigmaStat (Statcon). Continuous data was tested for normal distribution. If normality test failed, Mann-Whitney rank sum test was applied, otherwise Student's t-test was used. For categorical data, two-tailed Fisher's exact test was applied.

## Supporting Information

Figure S1 A, RT-PCR of the *syg-2* and *nephrin* rescue lines. *syg-2* is expressed in wild type animals and transgenic *syg-2* rescue lines, but not in the *syg-2(ky671)* mutants (upper panel). Nephrin is expressed only in the *nephrin* rescue lines (middle panel). *act-1* (ß-actin, lower panel) serves as control and is expressed equally in all strains. B-D, Quantification of worms with four or more ectopic SNB-1 clusters in the SSR. As none of the wild type worms had more than 3 synaptic vesicles in SSR, we set an arbitrary cut-off at 4 vesicle clusters and more, designated as the full blown mutant phenotype. B, Ectopic expression of *syg-2* or *nephrin* in the vulva epithelial cells significantly reduces the *syg-2(ky671)* mutant phenotype. 84% of the *syg-2(ky671)* worms displayed the full blown phenotype with more than 4 SNB-1 clusters in SSR, while only few of the ‘rescue’ strains displayed such a severe phenotype (16% of the *nephrin* expressing transgenic lines and 8% of the *syg-2* lines). There is no significant difference between *syg-2* and *nephrin* rescue lines. n>50 per strain. Fisher's exact test, two-sided. ***, p<0,001. Error bars, SEM. C, Expression of *syg-2* transgenes in HSN cannot rescue the *syg-1(ky652)* mutant defective synaptogenesis. 87% of *syg-1* mutant worms fell into the category of full blown phenotype with 4 or more ectopic synapses in SSR. Neither the *syg-2* rescue line nor the chimerical constructs composed of extracellular SYG-2 and intracellular SYG-1 showed significant reduction in SNB-1 punctae compared to the mutant worms. n>30 per strain. Fisher's exact test, two-sided. ***, p<0.001. n.s., not significant. Error bars, SEM. D, Expression of *syg-1* and *syg-1ex_syg-2in* in the vulva epithelial cells can reduce the *syg-2(ky671)* mutant phenotype. The reduction is slightly greater in the *syg-1ex_syg-2in* rescue lines, than in the *syg-1* rescue lines. n>50 per strain. Fisher's exact test, two-sided. ***, p<0,001. *, p<0,05. Error bars, SEM.
(TIF)
