# Supplementary figures and images for: Functional and Spatial Analysis of C. elegans SYG-1 and SYG-2, Orthologs of the Neph/Nephrin Cell Adhesion Module Directing Selective Synaptogenesis
Source: PLoS One. 2011 Aug 15;6(8):e23598. doi: 10.1371/journal.pone.0023598 (PMC3156230; doi:10.1371/journal.pone.0023598)

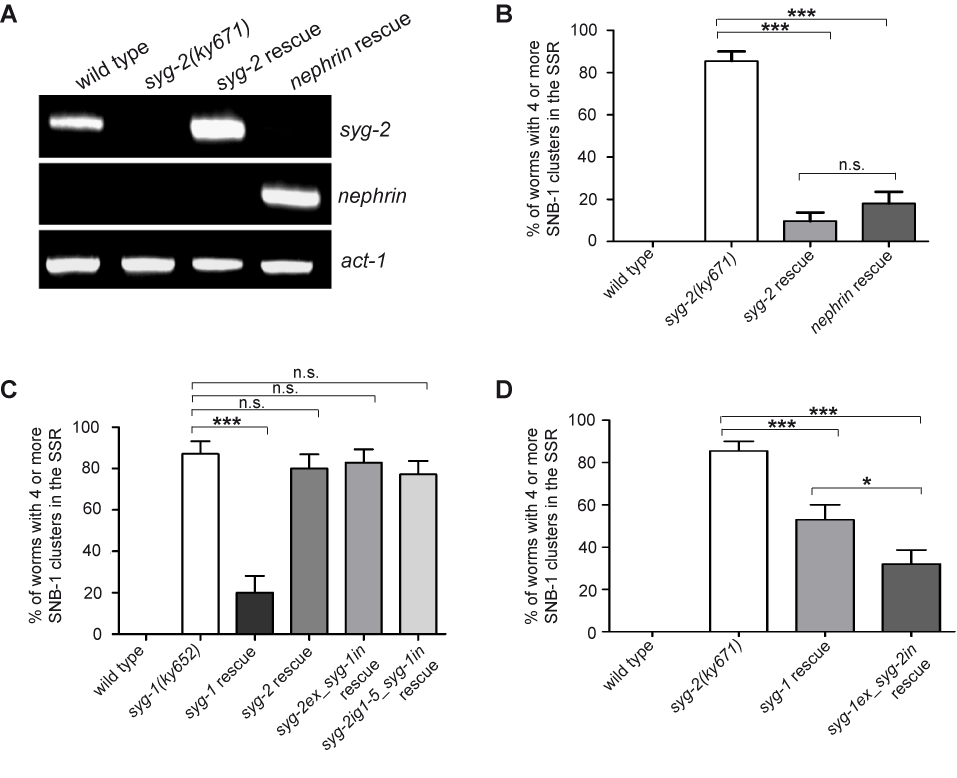

Supplement: Figure S1 — A, RT-PCR of the syg-2 and nephrin rescue lines. syg-2 is expressed in wild type animals and transgenic syg-2 rescue lines, but not in the syg-2(ky671) mutants (upper panel). Nephrin is expressed only in the nephrin rescue lines (middle panel). act-1 (ß-actin, lower panel) serves as control and is expressed equally in all strains. B-D, Quantification of worms with four or more ectopic SNB-1 clusters in the SSR. As none of the wild type worms had more than 3 synaptic vesicles in SSR, we set an arbitrary cut-off at 4 vesicle clusters and more, designated as the full blown mutant phenotype. B, Ectopic expression of syg-2 or nephrin in the vulva epithelial cells significantly reduces the syg-2(ky671) mutant phenotype. 84% of the syg-2(ky671) worms displayed the full blown phenotype with more than 4 SNB-1 clusters in SSR, while only few of the ‘rescue’ strains displayed such a severe phenotype (16% of the nephrin expressing transgenic lines and 8% of the syg-2 lines). There is no significant difference between syg-2 and nephrin rescue lines. n>50 per strain. Fisher's exact test, two-sided. ***, p<0,001. Error bars, SEM. C, Expression of syg-2 transgenes in HSN cannot rescue the syg-1(ky652) mutant defective synaptogenesis. 87% of syg-1 mutant worms fell into the category of full blown phenotype with 4 or more ectopic synapses in SSR. Neither the syg-2 rescue line nor the chimerical constructs composed of extracellular SYG-2 and intracellular SYG-1 showed significant reduction in SNB-1 punctae compared to the mutant worms. n>30 per strain. Fisher's exact test, two-sided. ***, p<0.001. n.s., not significant. Error bars, SEM. D, Expression of syg-1 and syg-1ex_syg-2in in the vulva epithelial cells can reduce the syg-2(ky671) mutant phenotype. The reduction is slightly greater in the syg-1ex_syg-2in rescue lines, than in the syg-1 rescue lines. n>50 per strain. Fisher's exact test, two-sided. ***, p<0,001. *, p<0,05. Error bars, SEM. (TIF) [file pone.0023598.s001.tif]
